# Mutagenicity of biodiesel or diesel exhaust particles and the effect of engine operating conditions

**DOI:** 10.7243/2050-1323-2-3

**Published:** 2013-03-09

**Authors:** Elena R Kisin, X.C Shi, Michael J Keane, Aleksandar B Bugarski, Anna A Shvedova

**Affiliations:** 1 Pathology and Physiology Research Branch, Health Effect Laboratory Division, National Institute for Occupational Safety and Health, Morgantown WV, USA.; 2 Exposure Assessment Branch, Health Effect Laboratory Division, National Institute for Occupational Safety and Health, Morgantown WV, USA.; 3 Office of Mine Safety and Health, National Institute for Occupational Safety and Health, Pittsburgh, PA, USA.

**Keywords:** Ames *Salmonella*/microsomal assay, mutagenic activity, biodiesel, diesel exhaust particulate matter, oxidation catalytic converter

## Abstract

**Background:**

Changing the fuel supply from petroleum based ultra-low sulfur diesel (ULSD) to biodiesel and its blends is considered by many to be a viable option for controlling exposures to particulate material (PM). This is critical in the mining industry where approximately 28,000 underground miners are potentially exposed to relatively high concentrations of diesel particulate matter (DPM). This study was conducted to investigate the mutagenic potential of diesel engine emissions (DEE) from neat (B100) and blended (B50) soy-based fatty acid methyl ester (FAME) biodiesel in comparison with ULSD PM using different engine operating conditions and exhaust aftertreatment configurations.

**Methods:**

The DPM samples were collected for engine equipped with either a standard muffler or a combination of the muffler and diesel oxidation catalytic converter (DOC) that was operated at four different steady-state modes. Bacterial gene mutation activity of DPM was tested on the organic solvent extracts using the Ames *Salmonella* assay.

**Results:**

The results indicate that mutagenic activity of DPM was strongly affected by fuels, engine operating conditions, and exhaust aftertreatment systems. The mutagenicity was increased with the fraction of biodiesel in the fuel. While the mutagenic activity was observed in B50 and B100 samples collected from both light-and heavy-load operating conditions, the ULSD samples were mutagenic only at light-load conditions. The presence of DOC in the exhaust system resulted in the decreased mutagenicity when engine was fueled with B100 and B50 and operated at light-load conditions. This was not the case when engine was fueled with ULSD. Heavy-load operating condition in the presence of DOC resulted in a decrease of mutagenicity only when engine was fueled with B50, but not B100 or ULSD.

**Conclusions:**

Therefore, the results indicate that DPM from neat or blended biodiesel has a higher mutagenic potency than that one of ULSD. Further research is needed to investigate the health effect of biodiesel as well as efficiency of DOC or other exhaust aftertreatment systems.

## Background

Biodiesel is considered an attractive alternative to diesel oil since it can be produced by domestic natural sources, reducing dependence on petroleum-based fuels [1]. Biodiesel can be produced from different plant oils by transesterification of triglycerides from vegetable oils with ethanol [2,3]. The increasing production and consumption of biodiesel has encouraged researchers to assess its hazard and fate in the environment. Specifically, diesel combustion engines are an important component of the industrial and transportation sectors, including numerous mining, agriculture and construction uses. There are concerns, however, with exhaust emissions in regards to potential adverse health effects associated with exposure to diesel particulate matter (DPM). Diesel engine emissions are highly complex mixtures of aerosols and gases. They consist of a wide range of organic and inorganic compounds [4]. Most of the particles from diesel engine exhausts are of nano-scale and therefore readily respirable. These particles potentially have hundreds of chemicals absorbed onto their surfaces, including known and suspected mutagens and carcinogens, *e.g.* polycyclic hydrocarbons (PAH) and nitrated PAH (nPAH) [5]. The formation of PAH depends on the type of engine, fuel composition, the engine operating conditions and the effectiveness of exhaust aftertreatment [6]. Exposure to diesel engine emissions and their atmospheric transformation products occur often in both environmental and occupational settings. Compared to their parent PAH, most of the resulting compounds generated from the combustion system are mutagens or have an enhanced mutagenic potency [7-9]. A causal relationship of exposure to diesel engine emissions and lung cancer was suggestive for occupational settings but not for the general population [5]. According to two large studies [10,11] conducted among the non-metal miners, diesel exhaust increases the risk of death from lung cancer. The International Agency for Research on Cancer [12], a part of the World Health Organization (WHO), classified diesel engine exhaust as carcinogenic to humans (Group 1) based on sufficient evidences to link exposure to an increased risk of lung cancer.

During recent years, strong efforts have been made to minimize diesel engine emission-related health hazards. This includes improved combustion, use of exhaust aftertreatment, the reduction of sulfur and aromatics content in fuels and lubricating oil, and the introduction of reformulated fuels [4,5]. Various diesel exhaust treatment technologies, such as diesel particulate filter (DPF) systems, disposable filters elements (DEDs), and diesel oxidation catalysts (DOCs) have been implemented. In mining, improvements of ventilation and the curtailment of diesel particulate matter (DPM) and toxic gaseous emissions from existing and new diesel powered equipment are commonly perceived as the most promising tools to meet Mine Safety and Health Administration (MSHA) regulations [4]. The use of biodiesel results in a substantial reduction of unburned hydrocarbons, carbon monoxide, and PM as compared to diesel emissions [13]. The issue of exposure to DPM and the use of biodiesel blends is particularly critical in the mining industry where approximately 28,000 U.S. underground miners are potentially exposed to relatively high concentrations of DPM [4]. Mine operators are currently using 25-100% biodiesel blends [14,15]. The concentrations of biodiesel in the blends used in underground mining are substantially higher than those used in other on- and off-highway applications [4].

The effects of biodiesel on emissions were found to vary widely, in particular with usage conditions, engine type and age [16-18]. Mine studies showed potential of neat biodiesel [18] and biodiesel blends [19-23] to reduce exposure of underground miners to DPM. However, the combustion of biodiesel in diesel engines typically results in slight increase of nitrogen oxide emissions [24]. The particle-bound volatile organic fractions of DPM [17,25] were found to be higher for biodiesel than for petroleum diesel fuels [17,25]. This may impact the biologic effects and toxicity of biodiesel exhaust particles.

Previous studies have compared the mutagenicity of diesel and biodiesel exhausts using bacterial reverse mutation assay [26,27]. This assay is suited as a screening tool for hydrophobic mutagenic compounds or mixtures, such as PAH from diesel engine emissions [5]. There is conflicting and limited information available regarding the mutagenic potential of biodiesel emissions specific to fuel category, engine type, and operating conditions [28-39].

The purpose of our study is to investigate the effects of soy-based fatty acid methyl ester (FAME) biodiesel, engine operating conditions and exhaust aftertreatment on the mutagenicity of diesel aerosol organic solvent extracts in *Salmonella typhimurium*. The samples analyzed in this study were collected directly in an underground mining environment of the National Institute for Occupational Safety and Health (NIOSH) Lake Lynn Experimental Mine by using the NIOSH Diesel Laboratory.

## Methods

### Sample collection

**Figure 1:**
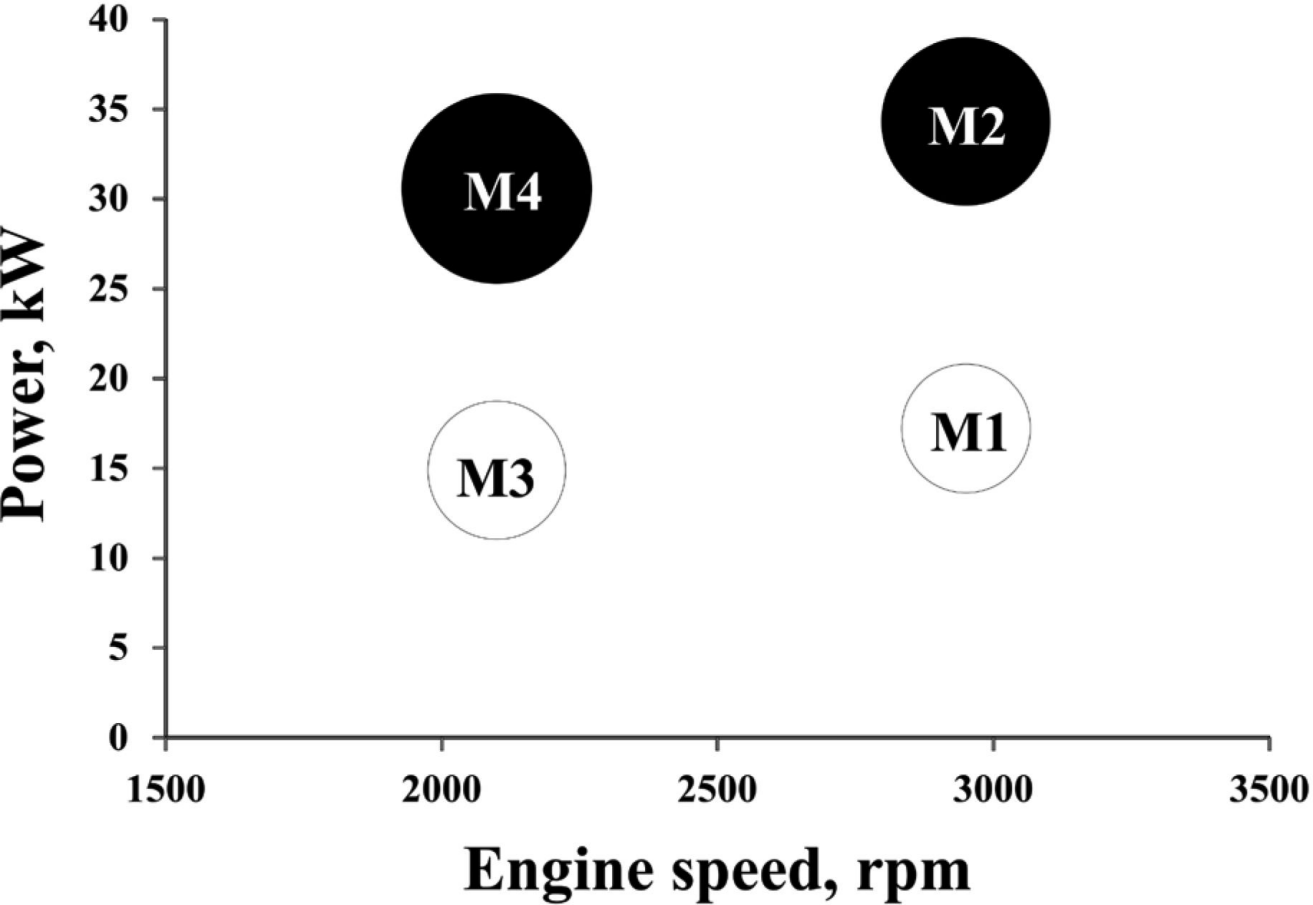
Engine operating conditions Engine was operated at four steady-state modes. One of the selected modes at each of the engine speeds represented heavy engine load (M2 and M4 with an engine speed of 2950 rpm and 2100 rpm, torque of 108.4 Nm and 138 Nm, and power 34.3 kW and 30.6 kW, respectively), while the other was more representative of light engine load (M1 and M3 with an engine speed of 2950 rpm and 2100 rpm, torque of 63.7 Nm and 73.2 Nm, and power 17.2 kW and 14.9 kW, respectively).

The DPM samples were collected at the diesel laboratory in the D-drift of the NIOSH Lake Lynn Experimental Mine. This laboratory was designed to allow evaluation of control technologies and strategies in an underground environment [23,40]. The tests were executed using Isuzu C240 engine rated at 41 kW, one of the most commonly used light-duty engine in US underground coal mines [41]. The engine was operated at four steady-state modes using eddy-current dynamometer (Figure 1). These modes were selected to cover a wide range of engine operating parameters. One of the selected modes at each of the engine speeds was representative of heavy engine loads (M2 and M4), while the other two were representative of light or medium engine loads (M1 and M3). Two exhaust configurations (muffler and muffler plus DOC) were evaluated for M1 and M2 modes. Soy-based FAME biodiesel (B100) was supplied by Stepan Company (Stepansol SB-W, Bordentown, NJ). ULSD supplied by Guttman Oil was used as baseline fuel. The B50 blend was prepared at the site. The fractions of biodiesel and ULSD were determined volumetrically. The DPM samples were collected concurrently on 90 mm Teflon coated glass fiber filters (Pall TX40HI20-WW). The nominal sampling flow rate for each of the sampling stream was 91 lpm. The sampling times were between 120 and 180 min depending on the test. The flow rates were maintained using rotary vane pumps (Gast, Model 1023) and controlled with mass flow controllers (Sierra Instruments Inc., Model 840). The Teflon-coated aluminum cyclone (URG, Model URG-2000-30EH) was used to remove aerosols with D_50_ larger than 1 μm. The properties of sampled aerosols are published in [23]. Eighteen samples were collected for mutagenicity analysis. Table 1 shows the information on the fuel, the exhaust configuration and engine operating modes pertinent to each of the samples.

### Samples preparation

Each sample consisted of DPM collected concurrently on two 90 mm filters. The DPM were extracted simultaneously from both filters using 100 ml of acetone and sonicated for 2 hours. The extract was reduced by evaporation in N_2_ environment and centrifuged at 4000 rpm for 40 minutes. The supernatant was removed and filtered through 25 mm sterile PTFE filter. The filtrate was transferred to a tared sterile brown vial and evaporated to dryness under N_2_. Twelve milligrams of DPM extract per milliliter of dimethyl sulfoxide (DMSO) was prepared as a stock solution. Samples were diluted further with DMSO to obtain the required concentration.

### Ames salmonella assay

The mutagenicity of samples was determined using the preincubation variant of the Ames Salmonella assay system [42]. Results from the previous studies indicate that *Salmonella typhimurium* strains TA98 and YG1024 gave better mutagenic response than TA100 and YG1029 to DPM tested, and S9 microsomal activation was not necessary for examination of mutagenic activity in DPM [43-45]. Due to the limited amounts of the samples available, only *Salmonella typhimurium* TA 98 without S9 microsomal activation was used in the study. The cell suspension of *Salmonella typhimurium* TA98 was prepared by overnight incubation of a small piece of frozen culture in 25ml nutrient broth. The genotype of the bacteria was confirmed following the recommendation by [42]. A sample solution of 10μl, along with 65μl physiological saline, and 25μl of the TA98 cultures were pre-incubated at 37°C for 30 min on a rotary drum incubator before plating. The control was established using DMSO in place of sample solution. After incubation, 2.5ml of molten top agar were added to each sample tube, the contents mixed, and poured onto a Vogel-Bonner minimal media plate. The plates were incubated at 37°C for 48h prior to counting. The conditions of the bacterial background lawn were examined during every experiment in order to know if cytotoxicity appeared in the high concentrations. Three readings of the revertant colonies per plate were scored by an automatic colony counter (Accucount 1000, Biologics Inc., Gainsville, VA) after incubation. The average numbers of revertant colonies per each concentration were calculated. An extract was considered mutagenic if the number of revertants in any of the concentrations tested was two-fold or greater than the control [27] and showed a dose related response. All experiments were repeated twice to confirm the results.

### Statistical analysis

Results were compared by One Way ANOVA using the All Pairwise Multiple Comparison Procedures (Holm-Sidak method). All results are presented as mean + standars error of the mean (SEM).

## Results

### Effects of B50 or B100 in comparison to ULSD

**Figure 2:**
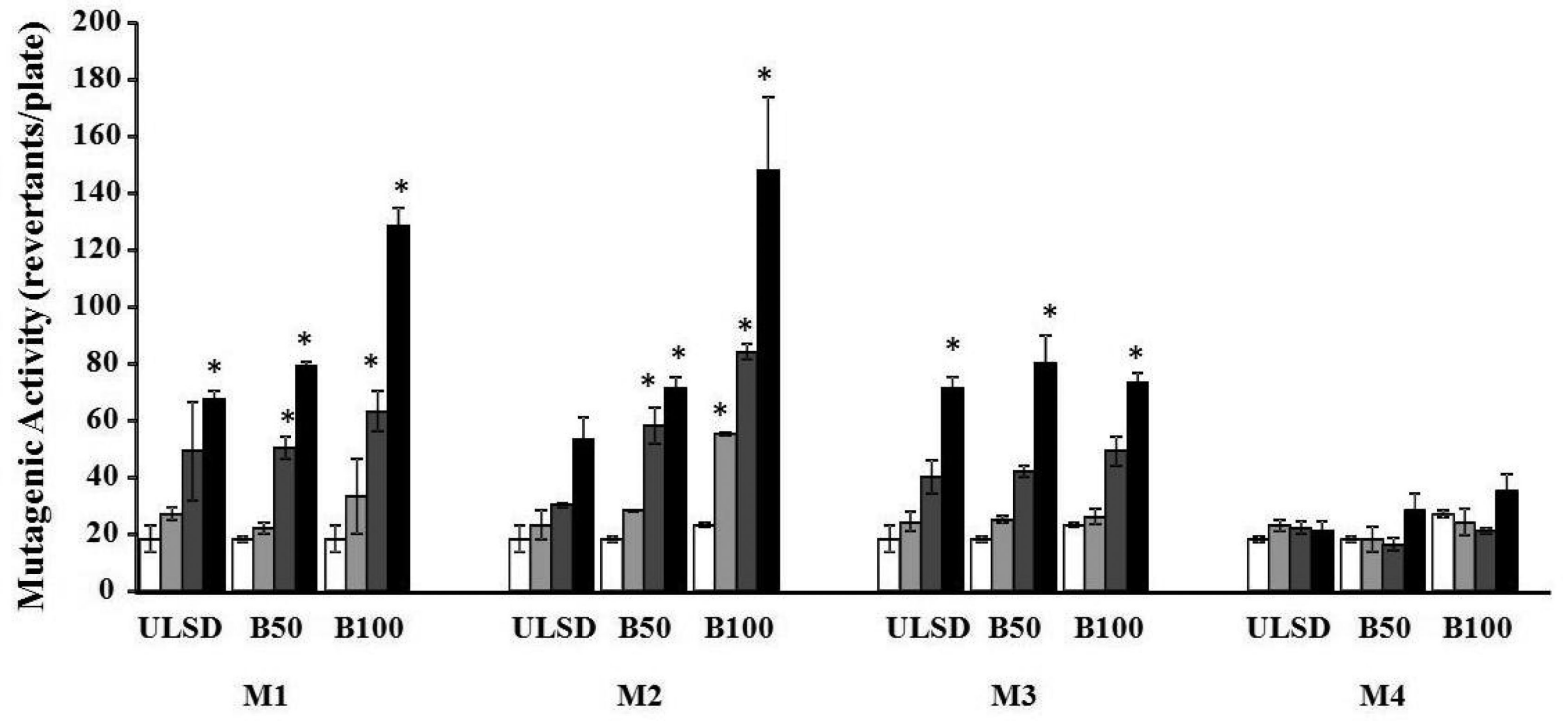
Mutagenic activity of DPM samples collected for the engine fueled with ULSD, B50 or B100 and equipped with muffler only at 4 steady-state modes ULSD, B50 or B100 samples (0, 13.3, 40 or 120 μg/plate) were incubated at 37° C with the *Salmonella typhimurium* TA 98 without S9 microsomal activation. Clear columns – control samples (PBS exposure); light gray columns – exposure with 13.3 μg/plate of DPM; Dark gray columns – exposure with 40 μg/plate of DPM; Black columns – exposure with 120 μg/plate of DPM. Data represent mean values (+SEM) of the average number of revertant colonies per sample. Each sample was tested twice in two separate experiments. *positive responses, as evidenced by the number of revertant colonies being at least two-fold greater than the respective control value.

The mutagenic effects of B50 and B100 were compared with those of the ULSD for all four tested engine conditions (Table 1, Figure 2). When engine was equipped with muffler, the concentration dependent mutagenic activity was observed for all samples collected at light-load (M1 and M3) and one of the heavy-load (M2) operating conditions. The mutagenic activity was increased with fraction of biodiesel in the fuel for all those 3 modes. B100 (120 μg/plate) induce the strongest mutagenic effect at M1 and M2 (91% and 179% over ULSD, respectively). At the same conditions, B50 caused only 18% and 34% increase in mutagenicity over ULSD, respectively. B50 and B100 did not induce a significant increase (13% and 3%, respectively) in mutagenic activity when engine was operated under M3 conditions, as compared to ULSD. No positive mutagenic activity was detected for all samples collected at M4 (heavy-load) operating conditions.

### Effect of engine operating conditions on mutagenic activity of DPM

**Figure 3:**
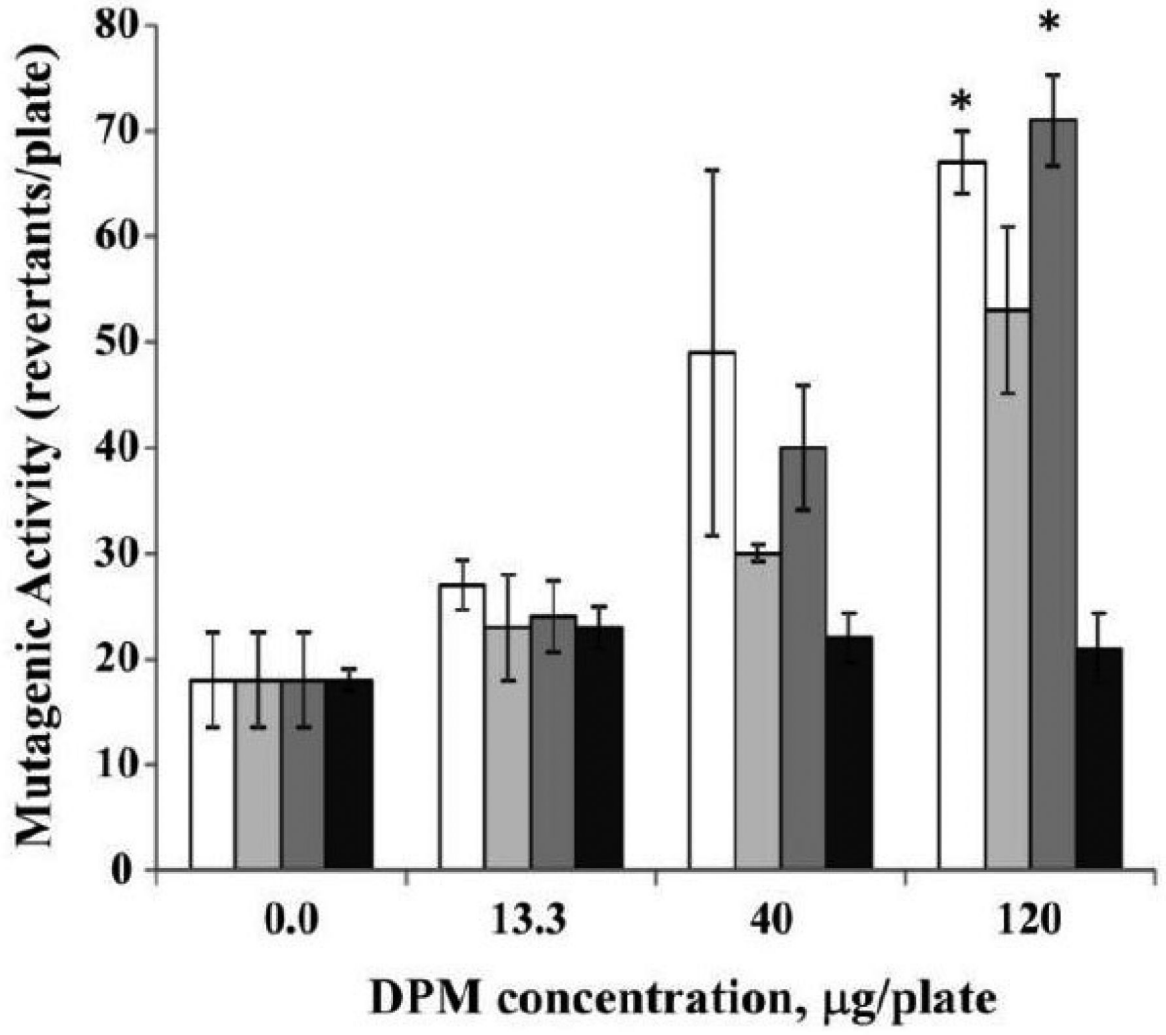
Effect of engine operating conditions on mutagenic activity of ULSD samples collected for the engine fitted with muffler ULSD samples (0, 13.3, 40 or 120 μg/plate) were incubated at 37° C with the *Salmonella typhimurium* TA 98 without S9 microsomal activation. Clear columns – mode M1; light gray columns – mode M2; Dark gray columns – mode M3; Black columns – mode M4. Data represent mean values (+SEM) of the average number of revertant colonies per sample. Each sample was tested twice in two separate experiments. *positive responses, as evidenced by the number of revertant colonies being at least two-fold greater than the respective control value.

**Figure 4:**
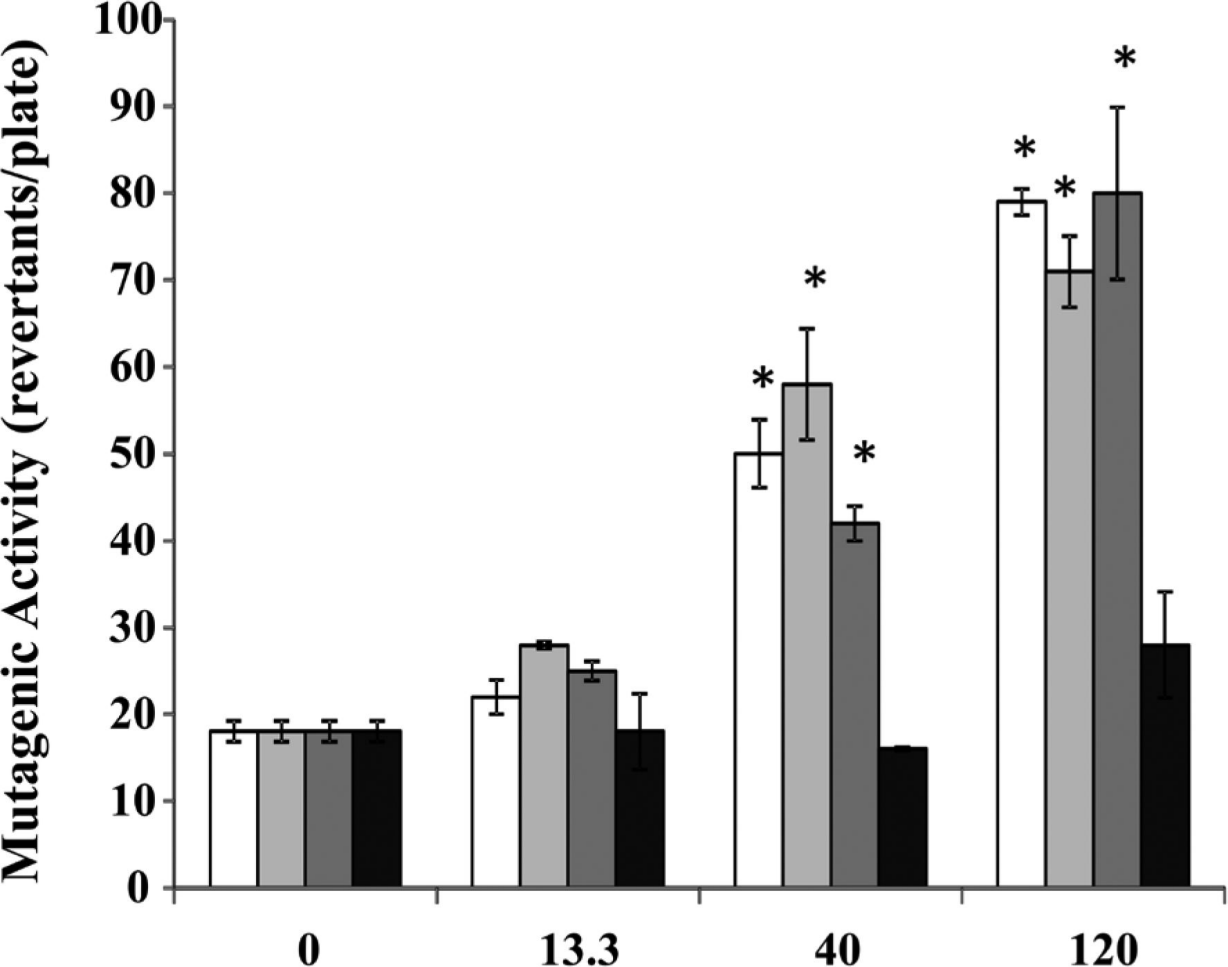
Effect of engine operating conditions on mutagenic activity of B50 samples collected for the engine fitted with muffler B50 samples (0, 13.3, 40 or 120 μg/plate) were incubated at 37° C with the *Salmonella typhimurium* TA 98 without S9 microsomal activation. Clear columns – mode M1; light gray columns – mode M2; Dark gray columns – mode M3; Black columns – mode M4. Data represent mean values (+SEM) of the average number of revertant colonies per sample. Each sample was tested twice in two separate experiments. *positive responses, as evidenced by the number of revertant colonies being at least two-fold greater than the respective control value.

**Figure 5:**
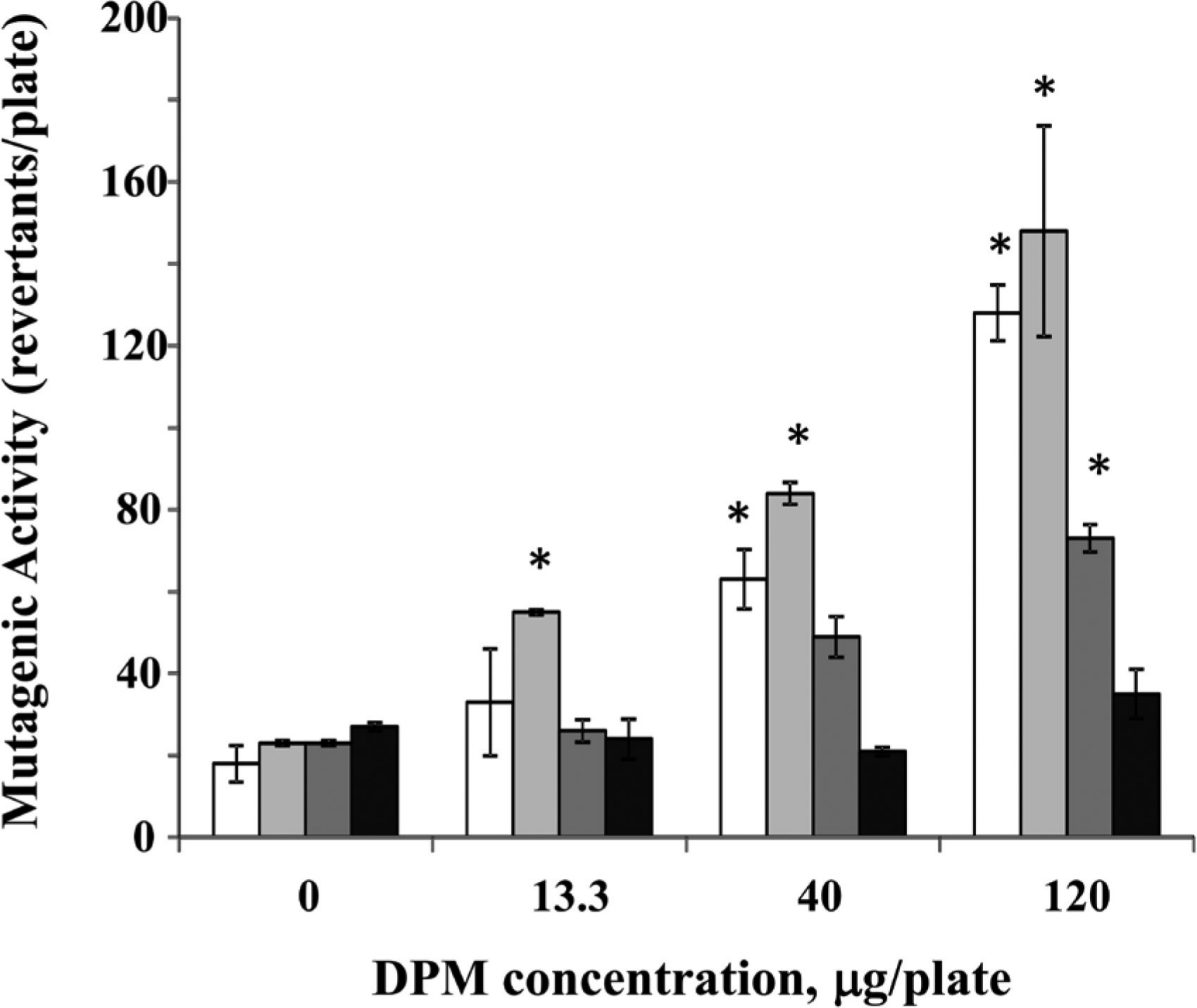
Effect of engine operating conditions on mutagenic activity of B100 samples collected for the engine fitted with muffler B100 samples (0, 13.3, 40 or 120 μg/plate) were incubated at 37° C with the *Salmonella typhimurium* TA 98 without S9 microsomal activation. Clear columns – mode M1; light gray columns – mode M2; Dark gray columns – mode M3; Black columns – mode M4. Data represent mean values (+SEM) of the average number of revertant colonies per sample. Each sample was tested twice in two separate experiments. *positive responses, as evidenced by the number of revertant colonies being at least two-fold greater than the respective control value.

The effects of the four engine operating conditions were studied using the samples collected from engine equipped with a muffler (Table 1 and Figures 3,4 and 5). When engine was fueled with ULSD, the mutagenic activity was observed for the samples collected for light-load engine operating conditions (M1 and M3), but not for heavy-load engine operating conditions (M2 and M4), and only for the highest studied concentration of 120 μg/plate (Figure 3). When engine was fueled with B50 (Figure 4) and B100 (Figure 5), the mutagenic activity was detected at both light-load (M1 and M3) and one of the heavy-load operating conditions (M2 but not M4). Increase in mutagenic activity was concentration dependent for all those 3 modes with the positive response at two highest concentrations (40 and 120 μg/plate). At the lowest studied concentration of 13.3 μg/plate, the mutagenic activity was found only for the engine fueled with B100 and operated at M2 condition (Figure 5).

### Effect of diesel oxidation catalyst (DOC) on mutagenic activity of DPM

**Figure 6:**
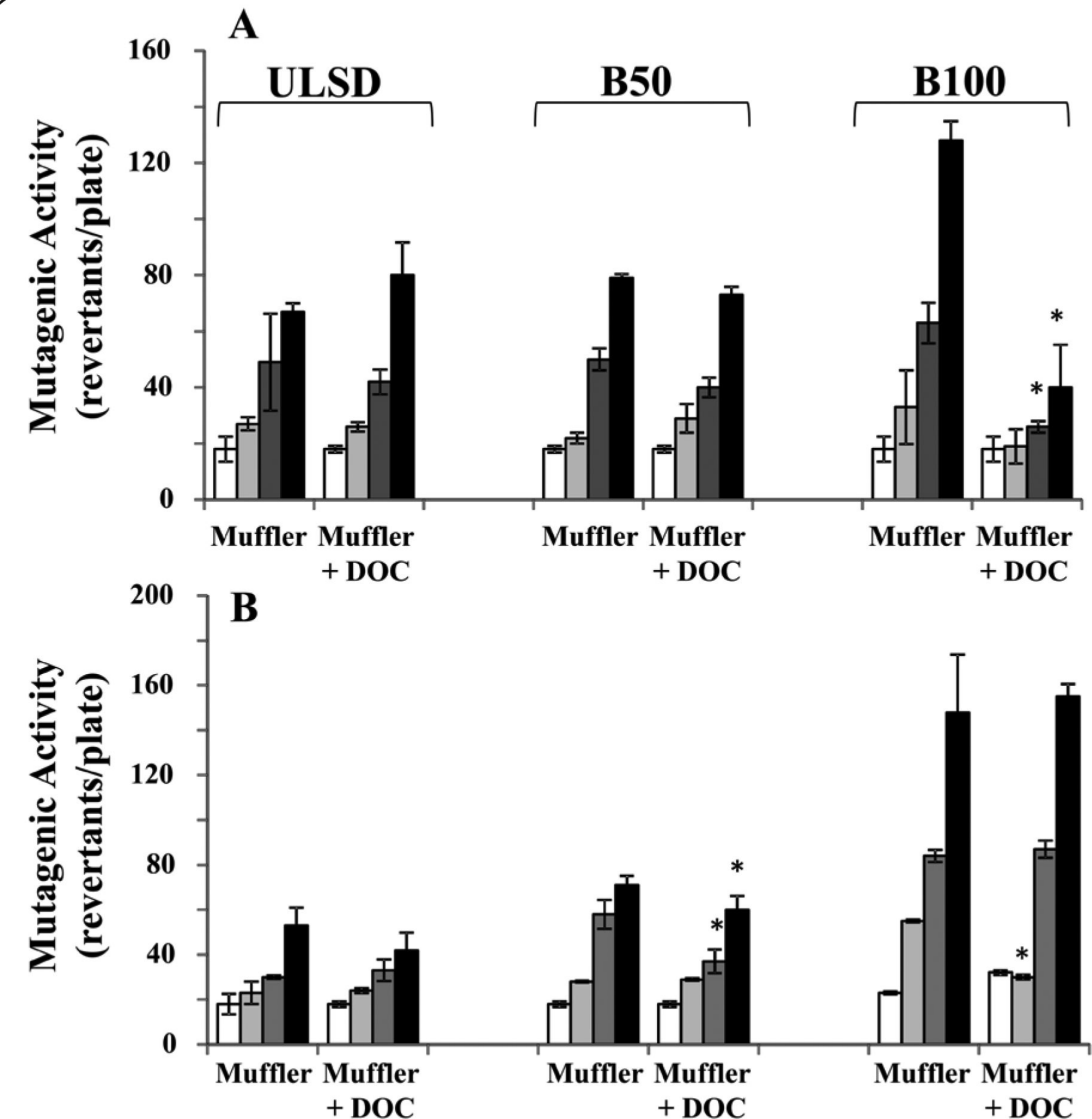
Effect of DOC on mutagenic activity of DPM samples collected for the engine operated at 2 steady-state modes M1 (A, light load) and M2 (B, heavy load) ULSD, B50 or B100 samples (0, 13.3, 40 or 120 μg/plate) were incubated at 37° C with the *Salmonella typhimurium* TA 98 without S9. Clear columns – control samples (PBS exposure); light gray columns – exposure with 13.3 μg/plate of DPM; Dark gray columns – exposure with 40 μg/plate of DPM; Black columns – exposure with 120 μg/plate of DPM. Data represent mean values (+SEM) of the average number of revertant colonies per sample. Each sample was tested twice in two separate experiments. *p<0.05, vs the engine equipped with muffler only.

The effects of DOC on mutagenic activity of DPM were studied using samples collected for all three fuels and the engine operated at one light-load (M1) and one heavy-load (M2) condition (Table 1, Figure 6). The strongest effects of DOC were observed when the engine was fueled with B100 and operated at mode M1 (Figure 6A). The results for two highest sample concentrations, 40 μg/plate and 120 μg/plate, showed that introduction of this specific DOC in the exhaust system resulted in 59% and 69% decrease in mutagenic activity, respectively. In the cases involving B100 and lowest sample concentrations (13.3 μg/plate), the samples collected for the engine equipped with DOC and operated at M2 exhibited 45.5% lower mutagenic activity than the corresponding sample obtained for the muffler-only configuration (Figure 6B). When the engine was fueled with B50, the effects of DOC were observed only for M2 conditions, demonstrating 12% and 16% decrease in mutagenic activity for two highest sample concentrations, respectively (Figure 6B). No significant effects of DOC were observed when the engine was fueled with ULSD.

## Discussion

This study was conducted to investigate the mutagenic effects of soy-based FAME biodiesel. The effects of B50 and B100 were compared to those of ULSD. These effects were examined for four steady state engine operating conditions and two exhaust aftertreatment configurations using *Salmonella typhimurium* TA98 (frame-shift mutation) assay without metabolic activation (S9).

This is well-known that emissions are influenced by both the engine (and exhaust aftertreatment system) as well as the fuel being combusted [46]. Accordingly, results of our study clearly indicate that mutagenic activity of DPM was strongly affected by the fuel formulation, engine operating conditions, and exhaust aftreatment. For the engine fueled with ULSD, only the DPM samples collected for light-load engine operating conditions were mutagenic. These results are consistent with the previous studies [45,46] and can possibly be explained by the lower brake thermal efficiency of the engine, less completed combustion, and consequently elevated emission of organic compounds at the light-load in comparison with the high-load engine operating conditions. In contrast, samples collected for the engine fueled with B50 or B100, the mutagenic activity was observed for both light-load and heavy-load engine operating conditions. In our study, the increase in mutagenic activity correlated with the increased level of the fraction of organic carbon in these samples [23]. Samples with the higher fraction of organic carbon also exhibited the higher genotoxic activity for the strain TA98 without S9 microsomal activation. For example, DPM samples obtained for M1 and M2 operating conditions had respectively 64% and 168% higher organic carbon content when B100 was used in place of ULSD [23]. Consequently, the B100 samples exhibited 91% and 179% higher mutagenic activity than corresponding ULSD samples. The DPM generated at heavy-load engine operating condition (M4) that was characterized with the lowest fraction of organic carbon of all samples collected [23] showed no positive mutagenic activity. The mutagenic activity increased with fraction of biodiesel in the fuel for three modes (M1-M3). Similarly, the results produced by the other groups [30,31], indicate a strong dependency of the number of revertants on the engine speed. In our study, the strongest increases in mutagenicity were triggered by B100 at the high-speed modes M1 and M2. In agreement with other studies [17,25], carbon analysis of the primary filter showed positive correlation between fraction of organic carbon and fraction of biodiesel in the fuel [23].

It is well established that particulate matter produced by combustion of petroleum diesel and biodiesel are a complex mixture of different compounds [5,48]. Regardless of the reduction in the total mass of particulate matter, the soluble organic fraction of the emitted particles is commonly found at a higher level in the exhaust of diesel engines fueled with biodiesel. Many studies have found that soluble organic fraction (SOF) of DPM contains polynuclear aromatic hydrocarbons (PAH) and nitro-PAH that are known to be genotoxic and probably are strong contributors to induce gene mutation in bacteria [9,49-52]. Some of the PAH measured in the DPM, like benzo(a)pyrene, fluoranthene or benzo(ghi)perylene (chemically reactive, non-volatile), emitted at the greater levels in the biodiesel than diesel exhaust [53]. These PAHs are known to be mutagenic and highly carcinogenic. The positive results without metabolic activation confirm that components like substituted PAH contribute significantly to mutagenic effects of DPM. This effect of direct acting mutagens is attributed to substituted PAH, such as nitro-PAH [5,54-56]. Nitro-PAH could be formed by a reaction with the NOx present in diesel engine emissions. These substances display a strong direct mutagenicity while there parent compounds exhibit no or less mutagenicity [5,8,57,58]. The combustion of biodiesel in a diesel engine usually increases the release of nitrogen oxides [59]. An increase in NO_2_ concentration in the B50 exhaust samples [23] corresponded with the higher mutagenicity observed for B50 samples as compared to ULSD. Furthermore, Biswas *et al.,* (2009) showed correlations between oxidative potential and organic carbon from DPM [60]. In our study we have an increased percentage of the organic carbon fraction for biodiesel samples in comparison with diesel exhaust. Higher concentrations of organic compounds and transition metals (Co, Cu, Ni, Zn) in the biodiesel exhaust than in diesel exhaust [61] might contribute to the higher oxidative potential of biodiesel DPM and thus to its higher *in vitro* mutagenicity. While the chemical characterization of the fuel exhaust is not part of the current study, future work focusing on deciphering the detailed compositions of the organic extracts of biodiesel and diesel exhaust and its effect on relation to the mutagenic activity, is highly prompted.

Benefits of using a DOC to control toxicity of DPM generates at light–and heavy-load engine operating conditions were investigated in our study. When the DOC was used, a strong correlation between engine operating conditions and observed changes in mutagenic activity of DPM was detected. DOC significantly decreased the mutagenic activity of DPM by 69% in the case of B100 and light-load (M1) engine operating conditions. A decrease in mutagenicity was also observed when engine equipped with DOC was fueled with B50 and heavily loaded. The decrease in mutagenicity can be explained by the fact that DOC effectively removed hydrocarbons and the organic carbon fraction of DPM [23]. Mutagenicity was slightly increased by the B100 exhaust (120 mg/plate) at high load mode M2 with a great increase in NO_2_ concentration [23], which might be responsible for synthesizing even more genotoxic materials, nitro-PAH, under these conditions. Our results are similar to those from the different studies showing that changes depended mainly on engine load modes [34,62,63]. They suggested that high engine load increases NOx in the exhaust and NOx cannot effectively be reduced by a DOC, while high NOx levels lead to an increased formation of nPAH.

## Conclusions

In conclusion, the neat and blended soy-based FAME biodiesel fuels demonstrated the potential to substantially reduce the mine air concentrations of elemental carbon (EC) and total DPM mass. However, the DPM generated by diesel engine fueled with neat or blended biodiesel was found to have higher mutagenic potency, as determined by Salmonella assays than DPM generated by the same engine fueled with ULSD. This increase in mutagenicity might be attributed to the higher presence of organic carbon in the biodiesel DPM. Since our results demonstrated that the evaluated DOC was effective in controlling OC level and mutagenic potential of DPM, we would recommend using optimized DOCs on the diesel engines particularly those fueled with biodiesel. Further research is needed to investigate the health effect of biodiesel exhaust as well as efficiency of the DOC and other exhaust aftertreatment devices in removal of specific potentially toxic species.

## Figures and Tables

**Table 1:** Mutagenicity induced by DPM samples

Exhaust Configuration	Engine Mode	Fuel	Revertants/plate^a^ in concentration (μg/plate)			
			0.0^b^	13.3	40.0	120.0
Muffler	Ml	**ULSD**	18±4.5	27±2.3	49±17.3	67±3.0^*^
Muffler	M2		18±4.5	23±5.0	30±0.8	53±7.9
Muffler	M3		18±4.5	24±3.4	40±5.9	71±4.3^*^
Muffler	M4		18±1.0	23±2.0	22±2.3	21±3.3
Muffler+DOC	Ml		18±1.2	26±1.6	42±4.4	80±11.7^*^
Muffler+DOC	M2		18±1.2	24±1.2	33±4.8	42±7.7
Muffler	Ml	**B50**	18±1.2	22±2.0	50±3.9^*^	79±1.5^*^
Muffler	M2		18±1.2	28±0.4	58±6.4^*^	71±4.1^*^
Muffler	M3		18±1.2	25±1.1	42±2.0^*^	80±9.9^*^
Muffler	M4		18±1.2	18±4.4	16±2.2	28±6.1
Muffler+DOC	Ml		18±1.2	29±5.1	40±3.5	73±2.8^*^
Muffler+DOC	M2		18±1.2	29±0.6	37±5.3	60±6.2^*^
Muffler	Ml	**B100**	18±4.5	33±13.1	63±7.2^*^	128±6.8^*^
Muffler	M2		23±0.6	55±0.6^*^	84±2.7^*^	148±25.7^*^
Muffler	M3		23±0.6	26±2.7	49±5.0	73±3.3^*^
Muffler	M4		27±1.1	24±4.9	21±1.1	35±6.0
Muffler+DOC	Ml		18±4.5	19±6.1	26±2.1	40±15.2
Muffler+DOC	M2		32±1.1	30±1.1	87±3.8^*^	155±5.5^*^

a Mean value of two plates ± standard deviation.

b Solvent control was shared within each group of samples assayed simultaneously.

* Positive response.
